# Assessment of Polycyclic Aromatic Hydrocarbon Contamination of Breeding Pools Utilized by the Puerto Rican Crested Toad, *Peltophryne lemur*

**DOI:** 10.5402/2012/309853

**Published:** 2012-12-16

**Authors:** Jenessa Gjeltema, Michael Stoskopf, Damian Shea, Ryan De Voe

**Affiliations:** ^1^Department of Clinical Sciences, College of Veterinary Medicine, North Carolina State University, 1060 William Moore Drive, Raleigh, NC 27607, USA; ^2^Environmental Medicine Consortium, College of Veterinary Medicine, North Carolina State University, 1060 William Moore Drive, Raleigh, NC 27607, USA; ^3^Department of Biology, North Carolina State University, 127 David Clark Labs, Raleigh, NC 27695-7617, USA; ^4^North Carolina Zoological Park, 4401 Zoo Parkway, Asheboro, NC 27205, USA

## Abstract

Habitat preservation and management may play an important role in the conservation of the Puerto Rican crested toad, *Peltophryne lemur*, due to this species' small geographic range and declining native wild population. Bioavailable water concentrations of Polycyclic Aromatic Hydrocarbon (PAH) contaminants within breeding pools at 3 sites were established using Passive Sampling Devices (PSDs) and gas chromatography-mass spectrometry (GC/MS). A more diverse population of PAH analytes were found in higher concentrations at the breeding site that allowed direct vehicular access, but calculated risk quotients indicated low risk to toad reproduction associated with the current PAH analyte levels.

## 1. Introduction

The Puerto Rican crested toad, *Peltophryne lemur*, is the only native toad species of Puerto Rico and has become a subject of conservation concern due to its small population size, limited breeding sites, and small geographic range [1–3]. Although several new populations have been established in Puerto Rico through captive-breeding and release programs, reproduction of the naturally wild population of this toad species is considered limited to a small region of coastline located in Guanica, Puerto Rico [4–8]. Based on direct observation of breeding events during heavy rainfall, the number of observed mature individuals declined from 1984 to 2003, with only 80 mature individuals recorded in 2003 [1, 9]. The majority of breeding for the naturally wild population is thought to occur at three distinct breeding sites within a several kilometer radius, and each of these three breeding sites contains at least one ephemeral pool that fills with water under adequate rainfall conditions [2]. The volume, surface area, depth, and duration of each of these temporary pools is dependent on the amount and frequency of rainfall that the region receives. A portion of the largest and most significant site for toad reproduction, the Tamarindo site, is accessible for vehicular parking by members of the public when the breeding pools are dry. This parking occurs directly over areas where breeding pools form when it rains. 

Vehicular traffic is associated with a wide variety of contaminants including Polycyclic Aromatic Hydrocarbons (PAHs) from incomplete combustion, exhaust, oil leaks, tire abrasion, asphalt, and other lubricants [10–14]. PAHs have been linked with many undesirable health consequences in humans and animals including carcinogenic, immunotoxic, mutagenic, and teratogenic effects [15–18]. Environmental exposure of amphibians to PAHs may cause such broad effects as increased mortality, genotoxicity, larval deformities, histological changes to the integument, slowed development, and larval hyperactivity [11, 19–25]. A more thorough review and discussion of the effect of PAHs and other contaminants on amphibian populations may be found elsewhere [17, 26, 27].

 Passive sampling has been used for several decades as a method to assess contaminant levels within the air and aquatic environments. Passive Sampling Devices (PSDs) allow time-weighted average concentrations of contaminants to be quantified giving a better understanding of the exposure than random discrete water testing. Because the devices accumulate contaminants over a deployment period, passive sampling technology is often able to detect trace levels of contaminants in an aquatic environment better than other methods. The Semipermeable Membrane Device (SPMD) is a common configuration, which is usually composed of low-density polyethylene (LDPE) tubing containing a neutral lipophilic triolein core [32]. The LDPE tubing forms transient thermal pores to a size that closely approximates those of aquatic organisms [32, 33]. When such SPMDs are deployed in an environment containing lipophilic contaminants, such as PAHs, these analytes diffuse through the membrane to become sequestered within the core or membrane itself. This ensures that only the bioavailable form of the analyte is accumulated within the device and mimics the uptake of contaminants from the environment by live organisms. Following a deployment period, these contaminants can be extracted from the device using a nonpolar solvent and analyzed using gas chromatography-mass spectrometry (GC/MS) to identify and quantify the contaminants present within the device. Modeling can then be used to determine the concentrations of these contaminants within the aquatic environment [32, 34–37].

Current efforts to conserve the Puerto Rican Crested Toad are being made through captive breeding, reintroduction programs, habitat conservation, and public education conducted by the Puerto Rican crested toad Species Survival Plan [4, 5, 8, 9]. Habitat preservation may play a critical role in the survival of the remaining naturally wild population of toads. Contaminant levels at high enough concentrations could pose a significant risk to the development and survival of offspring within the breeding pools utilized by this species. This study was conducted to measure and perform a risk analysis of the bioavailable concentrations of PAHs present within breeding pools utilized by Puerto Rican crested toads at sites used for vehicular parking compared to those that are not.

## 2. Materials and Methods

### 2.1. Materials

The PSDs used in this study were composed of a single strip of dichloromethane-extracted LDPE tubing with the dimensions of 25 cm × 7.5 cm, an effective surface area of 375 cm^2^, and an average weight of 4.3 g. During deployment, these strips were housed between two rectangular fenestrated aluminum plates with the dimensions of 30 cm × 9 cm, which were curved along the longitudinal axis to form a cylindrical housing for the device. Six small slices were made along the edges of each LDPE strip to allow conventional plastic ties to hold the strip in place within the aluminum plates. These plates allowed the strips to be positioned at their respective deployment sites, provided shade, and elevated the strips above the sediment and within the water column. Small plastic placards were placed on each device and secured with plastic ties to deter theft during the deployment period. The components of the devices were stored wrapped in baked aluminum foil at −20°C until the time of the deployment. 

### 2.2. Deployment and Recovery

Deployment of the devices was scheduled for the first adequate rainfall of the rainy season, which occurred on November 8, 2009. Concurrent deployment of the devices was initiated on November 14-15, 2009. Recovery of the devices was performed 14 days following the initial deployment of each device to ensure that analytes remained in the linear uptake phase for appropriate modeling purposes. Devices were deployed into the ephemeral pools located at the three breeding sites: Tamarindo, Aroma, and Atolladora. Device placement was based on the specific pool configuration at each site favoring deep depressions to ensure that devices were not exposed to air as the pools evaporated during the deployment period. 15 devices were deployed at the Tamarindo site and were divided between its two major pools. 8 of these devices were deployed at the Tamarindo South pool, and 7 devices were deployed at the Tamarindo North pool. 6 devices were deployed at the Aroma site, and 8 devices were deployed at the Atolladora site. GPS locations were recorded for each device to ensure recovery. 

Following the deployment period, the LDPE strips were removed from their aluminum plates and rinsed in the water at their respective deployment locations to remove any organic debris. Each strip was wrapped individually in aluminum foil and placed in a sealed plastic bag inside a cooler containing ice packs for several hours until the devices could be stored below −20°C, which has been validated as an appropriate storage technique for SPMDs for periods up to 6 months [35]. The LDPE strips remained under these storage conditions until analysis, which was performed several weeks following recovery of the devices. The pool located at the Aroma site sustained significant natural volume loss during the deployment period, causing all of the devices at this site to become exposed to air prior to recovery. The devices from this site were not analyzed and were removed from the remaining aspects of this study.

Nitrile gloves were worn during deployment and recovery of the devices and were changed between the handling of each device. To ensure quality control, a separate field blank for each site was exposed to the air during deployment and recovery of the devices at each respective site. These field blanks were stored identically to the other devices prior to and after the deployment period and were stored wrapped in aluminum foil within a sealed plastic bag at −20°C throughout the deployment period.

### 2.3. Sample Analysis

To prepare the samples for analysis following recovery, each LDPE strip was allowed to thaw and then scrubbed under deionized water to remove any residual algae and organic matter. There was no significant biofouling observed on any of the strips. Each strip was cut into pieces of 1 cm by 4 cm with solvent-rinsed scissors. These pieces were placed into solvent-rinsed and labeled containers and were extracted twice over 24 hours using a total of 45 mL of dichloromethane. Gel permeation chromatography was used to fractionate the waxes and analytes of these samples using the technique previously described [37]. A procedural blank was prepared and extracted concurrently with the samples to ensure quality control. 

Between 4 and 7 samples for each site were analyzed using an Agilent 6890 gas chromatograph with 5973N mass selective detector. The column used was a Varian VF-5MS 30 m × 0.25 mm id (0.25 *μ*m film thickness), with 10 m integrated guard column. The initial temperature used was 40°C for 1 minute and then increased 25°C per minute until a temperature of 100°C. Following this, a temperature program of 5°C per minute to a final temperature of 310°C was used with a hold time of 15 minutes. The inlet temperature was held at 300°C, and a pulsed splitless injection technique was used. The inlet pressure was increased to 30 psi for 0.9 minutes and then reduced to maintain a constant column flow of 1 mL/min. The transfer line of the mass selective detector was held at 300°C and was operated in selected ion monitoring mode for analysis.

Perdeuterated surrogate recoveries for naphthalene d-8 from each of the devices ranged from 39% to 69% with the procedural control having a recovery of 80%. Recovery of acenaphthene d-10 ranged from 42% to 74% with the procedural control having a recovery of 74%. The recovery for chrysene d-12 ranged from 85% to 99% with the procedural control having a recovery of 103%. The recovery for perylene d-12 ranged from 65% to 71% with the procedural control having a recovery of 82%.

### 2.4. Modeling

The linear uptake model was used to derive water concentrations for each pool using the measured amount of analyte recovered from a specific device, field data, and a laboratory-derived sampling rate for that analyte [32]. The following equation was used to calculate the water concentrations of specific analytes in this study, and a full description of the model and the derivation of this equation are described elsewhere [32, 34–37]

(1) $$\begin{array}{c} C_{W}=\frac{N_{\text{PSD}}}{R_{S}}\times t. \end{array}$$

In this equation, *C*_*W*_ is the concentration of the contaminant or analyte in the water (ng/L), *N*_PSD_ is the amount of analyte sorbed by the device (ng), *R*_*S*_ is the laboratory derived sampling rate (*L*/*d*), and *t* is the length of the deployment (*d*). In order to account for differences between the configuration of the devices used in this study compared to those used for the laboratory derived sampling rates, these rates were adjusted by a factor of 0.833 [37].

### 2.5. Traffic Assessment

To assess vehicular traffic density at each site, parking data was taken during the period of July 13–29, 2009. GPS locations over the engine compartment of every car parked in the vicinity of the Atolladora, Aroma, and Tamarindo sites were taken three times per day with data collection not occurring at an interval shorter than two hours from the commencement of the previous data collection. The number of cars parked within a 100 meter buffer zone of the estimated center of each site was measured. The estimated center of each buffer zone was based upon the central location of the largest breeding pool present at each site. The specific GPS locations were redacted from this study due to conservation concerns. The GPS unit used to collect data for assessment of parking density was the Garmin GPSMAP 60CSx model. This GPS unit has a typical position accuracy of less than 10 meters [38]. ArcGIS software was used to perform the analysis of all traffic data collected.

### 2.6. Risk Analysis

To assess the risk associated with the analytes measured at each site, risk quotients were created for each analyte by calculating a ratio of the average water concentration at each site to the published water quality criteria [39]. Water quality criteria were obtained from several sources including the EPA's National Recommended Water Quality Criteria, Canadian Water Quality Guidelines for the Protection of Aquatic Life, British Columbia Water Quality Guidelines, Toxicological Benchmarks for Aquatic Biota from the US, Department of Energy, and toxicity studies from the primary literature. In the event that multiple water quality criteria were published for a single analyte, the most stringent value was used to calculate the risk quotient.

## 3. Results and Discussion

### 3.1. Traffic Assessment

The traffic analysis revealed that the average number of cars parked within a 100-meter buffer zone from the center of the Tamarindo South site was 17.56 cars. This is almost double the number of cars recorded for the Tamarindo North and Atolladora sites, which averaged at 9.29 and 9.13 cars, respectively. It is important to note that the Tamarindo South site and location of its breeding pool was directly accessible and frequently used for vehicular traffic and parking. Vehicular access was not permitted at the Tamarindo North and Atolladora sites; however, vehicles did use adjacent parking and roadways.

### 3.2. Derived Water Concentrations of PAH Analytes

The average derived water concentrations for analytes present within the ephemeral pools of each breeding site are listed in Table 1. Data for the procedural and field blanks for each site are also included. Biphenyl, C1-naphthalenes, fluorene, C1-C3 dibenzothiophenes, phenanthrene, anthracene, 1-methylphenanthrene, C1-C3 phenanthrenes/anthracenes, fluoranthene, pyrene, C1-fluoranthenes/pyrenes, retene, benz[a]anthracene, chrysene, C1-C3 chrysenes, and benzo[e]pyrene were all found to be in higher concentrations than combined field blank and procedural blank concentrations for the Tamarindo South site. Naphthalene, biphenyl, 2,3,5-trimethylnaphthalene, C2 naphthalene, C1-2 phenanthrenes/anthracenes, fluoranthene, pyrene, and retene were all found in higher concentrations than background levels at the Tamarindo North site. Phenanthrene, 1-methylphenanthrene, C1-2 phenanthrenes/anthracenes, fluoranthene, pyrene, C1-fluoranthenes/pyrenes, and retene were found to be above background levels at the Atolladora site.

The Tamarindo South site had a more diverse population of PAH analytes present within its breeding pool. 22 out of the 48 PAH analytes examined were found to be above background levels at the Tamarindo South site. Only 9 and 8 of the PAH analytes were found to be above background levels at the Tamarindo North and Atlladora sites, respectively. The Tamarindo South site also had higher concentrations of almost every analyte measured above background levels compared to the other two sites with the exclusion of naphthalene, biphenyl, C-2 naphthalenes, and 2,3,4-trimethylnaphthalene. These 4 analytes were found in slightly higher concentrations at the Tamarindo North site. 

### 3.3. Risk Analysis

The overall risk as indicated by the risk quotients depicted in Table 2 is negligible for most of the analytes measured. Chrysene, benz[a]anthracene, and pyrene at the Tamarindo South site represent those analytes with the highest risk with quotients of 0.226, 0.022, and 0.021, respectively. As indicated by the calculated quotients, the risk associated with the current concentrations of PAH analytes present in the breeding pools appears to be very low based on published water quality criteria. Several of the published water quality criteria are based on standards for human consumption and do not necessarily reflect the potential exposure that submerged toad embryos or tadpoles may encounter. Criteria designed for human consumption may not reflect the unique physiologic adaptations specific to amphibians that could alter the sensitivity, metabolism, and ultimate effect of analytes in this class of animal. Additionally, several analytes present within these breeding pools had no published water quality criteria data, and risk quotients could not be calculated for these analytes. 

## 4. Conclusion

This study was conducted to assess the bioavailable concentrations of PAH analytes present within three breeding pools utilized by Puerto Rican crested toads at sites used for direct vehicular parking compared to those that are not. A more diverse population of PAH analytes were found in higher concentrations within the Tamarindo South breeding pool, which is associated with higher levels of vehicular activity than the other two sites. Risk analysis for each site indicated low-risk quotients for the current concentrations of PAH analytes found at all three sites based on published water quality criteria. Interpretation of the risk analysis is confounded by the use of several water quality criteria based on human consumption standards as well as a lack of published criteria for some of the analytes found within the breeding pools. This study was limited to the assessment of PAH analyte concentrations and does not preclude the possibility that other organic or inorganic contaminants may be affecting toad reproduction at these sites.

## Figures and Tables

**Table 1:** Average derived water concentration of PAH analytes by breeding site.

Analyte	Procedure and field blanks			Tamarindo South		Tamarindo North		Atolladora	
	Procedural blank	Tamarindo field blank	Atallodora field blank	Range	Mean (std. dev.)	Range	Mean (std. dev.)	Range	Mean (std. dev.)
					*n* = 5		*n* = 5		*n* = 7
	ng/L	ng/L	ng/L	ng/L	ng/L	ng/L	ng/L	ng/L	ng/L
Naphthalene	0.07		0.07	0.00–0.10	0.05 (0.04)	0.06–0.15	0.11 (0.04)	0.00–0.07	0.06 (0.03)
2-Methylnaphthalene	0.01	0.02	0.03	0.02–0.05	0.03 (0.01)	0.02–0.03	0.03 (0.01)	0.02–0.03	0.02 (<0.005)
1-Methylnaphthalene	<0.005	0.01	0.01	0.01–0.03	0.02 (0.01)	0.01–0.02	0.01 (0.01)	0.02–0.02	0.02 (<0.005)
Biphenyl				0.00–0.03	0.01 (0.01)	0.00–0.03	0.02 (0.01)		
2,3,5-Trimethylnaphthalene						0.04–0.06	0.04 (0.01)		
C1-Naphthalenes	0.01	0.03	0.04	0.04–0.08	0.05 (0.02)	0.03–0.08	0.05 (0.02)	0.04–0.05	0.05 (0.01)
C2-Naphthalenes						0.00–0.14	0.08 (0.07)		
Fluorene			0.04	0.00–0.03	0.02 (0.01)			0.00–0.03	0.01 (0.01)
C1-Dibenzothiophene				0.18–0.42	0.25 (0.10)				
C2-Dibenzothiophene				0.00–2.00	0.78 (0.74)				
C3-Dibenzothiophene				0.00–4.70	1.49 (1.92)				
Phenanthrene		0.13	0.14	0.18–0.33	0.24 (0.05)	0.13–0.33	0.13 (0.02)	0.16–0.17	0.16 (0.01)
Anthracene				0.00–0.03	0.01 (0.02)				
1-Methylphenanthrene		0.02	0.02	0.09–0.22	0.14 (0.05)	0.02–0.22	0.02 (0.02)	0.03–0.05	0.03 (0.01)
C1-Phenanthrenes/anthracenes		0.11	0.10	0.49–1.08	0.71 (0.23)	0.10–1.08	0.16 (0.09)	0.14–0.16	0.15 (0.01)
C2-Phenanthrenes/anthracenes		0.18	0.25	0.98–3.18	1.59 (0.90)	0.18–3.18	0.25 (0.14)	0.22–0.35	0.26 (0.04)
C3-Phenanthrenes/anthracenes				0.00–4.46	1.68 (1.84)				
Fluoranthene				0.30–0.79	0.46 (0.20)	0.00–0.13	0.08 (0.05)	0.12–0.14	0.13 (0.01)
Pyrene				0.25–0.81	0.41 (0.23)	0.05–0.07	0.06 (0.01)	0.07–0.08	0.07 (<0.005)
C1-Fluoranthenes/pyrenes				0.30–1.07	0.5 (0.32)			0.00–0.05	0.01 (0.02)
Retene				0.10–0.28	0.15 (0.08)	0.09–0.14	0.11 (0.02)	0.05–0.10	0.07 (0.02)
Benz[a]anthracene				0.07–0.16	0.09 (0.04)				
Chrysene				0.55–1.85	0.86 (0.56)				
C1-Chrysenes				0.51–1.93	0.85 (0.61)				
C2-Chrysenes				0.54–1.87	0.86 (0.57)				
C3-Chrysenes				0.00–1.12	0.22 (0.50)				
Benzo[e]pyrene				0.00–0.93	0.30 (0.38)				

2,5-dimethylnaphthalene, acenaphthylene, acenaphthene, dibenzofuran, C2-C4-naphthalenes, 1-methylfluorene, C1-3-fluorenes, dibenzothiophene, C4-phenanthrenes/anthracenes, C4-chrysenes, benzo[b]fluoranthene, benzo[k]fluoranthene, benzo[a]pyrene, perylene, indeno[1,2,3-cd]pyrene, dibenz[a,h]anthracene, benzo[g,h,i]perylene, and coronene were measured below detectable limits for all sites.

**Table 2:** Risk assessment for analyte concentrations by site.

Analyte	Risk quotient for Tamarindo South	Risk quotient for Tamarindo North	Risk quotient for Atolladora	Water quality criteria (ug/L)	Note	Reference
Napthalene	<0.005	<0.005	<0.005	1	Freshwater, chronic	[28]
2-Methylnapthalene	<0.005	<0.005	<0.005	330	Freshwater, chronic	[3]
1-Methylnapthalene	<0.005	<0.005	<0.005	2.1	Freshwater, chronic	[29]
Biphenyl	<0.005	<0.005		14	Freshwater, chronic	[29]
Fluorene	<0.005		<0.005	3	Freshwater	[30]
Phenanthrene	<0.005	<0.005	<0.005	0.3	Freshwater, phototoxic	[28]
Anthracene	<0.005			.012	Freshwater	[30]
Fluoranthene	0.011	<0.005	<0.005	0.04	Freshwater	[30]
Pyrene	0.021	<0.005	<0.005	0.02	Freshwater, phototoxic	[28]
Benz[a]anthracene	0.022			0.004	Human	[31]
Chrysene	0.226			0.004	Human	[31]

Specific water quality criteria were not available for 2,3,5-trimethylnaphalene, C1-C2 naphthalenes, C1-3 dibenzothiophene, 1-methylphenanthrene, C1-3 phenanthrenes/anthracenes, C1-fluoranthenes/pyrenes, retene, and C1-2 chrysenes.
